# Dendritic Pyridine–Imine Copper Complexes as Metallo-Drugs

**DOI:** 10.3390/molecules29081800

**Published:** 2024-04-16

**Authors:** Régis Laurent, Valérie Maraval, Vania Bernardes-Génisson, Anne-Marie Caminade

**Affiliations:** 1 Laboratoire de Chimie de Coordination du CNRS, 205 Route de Narbonne, BP 44099, 31077 Toulouse, CEDEX 4, France; regis.laurent@lcc-toulouse.fr (R.L.); valerie.maraval@lcc-toulouse.fr (V.M.); vania.bernardes-genisson@lcc-toulouse.fr (V.B.-G.); 2 LCC-CNRS, Université de Toulouse, CNRS, 31077 Toulouse, France

**Keywords:** dendrimer, Schiff base, pyridine–imine, copper, metallo-drugs, cancer, phosphorus, carbosilane, PAMAM

## Abstract

Since the discovery of cisplatin in the 1960s, the search for metallo-drugs that are more efficient than platinum complexes with negligible side effects has attracted much interest. Among the other metals that have been examined for potential applications as anticancer agents is copper. The interest in copper was recently boosted by the discovery of cuproptosis, a recently evidenced form of cell death mediated by copper. However, copper is also known to induce the proliferation of cancer cells. In view of these contradictory results, there is a need to find the most suitable copper chelators, among which Schiff-based derivatives offer a wide range of possibilities. Gathering several metal complexes in a single, larger entity may provide enhanced properties. Among the nanometric objects suitable for such purpose are dendrimers, precisely engineered hyperbranched macromolecules, which are outstanding candidates for improving therapy and diagnosis. In this review article, we present an overview of the use of a particular Schiff base, namely pyridine–imine, linked to the surface of dendrimers, suitable for complexing copper, and the use of such dendrimer complexes in biology, in particular against cancers.

## 1. Introduction

Since the discovery of cisplatin in the 1960s and the success of its use to treat cancers [1], researchers in bioinorganic chemistry have first focused on platinum derivatives, which are of current use to treat testicular, ovarian, and lung cancers [2]. The search for metallo-drugs more efficient than platinum complexes, while having negligible side effects, has attracted much interest, as recently emphasized in a review of *in vivo* studies of the anticancer activities of metal complexes [3]. Among the metals that have been thoroughly examined for potential applications as anticancer agents are mainly ruthenium, titanium, gold, and copper. Some of the corresponding complexes have been designed for clinical trials [4,5]. The use of copper complexes as anticancer agents has been reviewed early [6] in an article structured according to the type of ligands used to complex copper [7]. The interest in copper was boosted in the last few years, on the one hand, by the hypothesis that an endogenous metal might exhibit fewer adverse effects on the body than exogenous metals, such as platinum, and on the other hand, by the discovery of cuproptosis, a recently evidenced form of cell death mediated by copper, which has potential applications in the treatment of cancer [8,9,10,11], with the aim of overcoming anticancer drug resistances [12]. However, copper is also known to induce the proliferation of cancer cells, as it stimulates the proliferation and migration of endothelial cells, as well as numerous pro-angiogenic reactions [13], which are the first stages of tumor angiogenesis [14]. It has been shown, in particular, that serum copper concentration increases as the cancer disease progresses [15]. In view of these contradictory results [16,17], there is a need to find the most suitable copper chelators [18] with the aim of reaching copper homeostasis [19,20]. Among these chelators, Schiff-based derivatives offer a wide range of possibilities [21], particularly for the biomedical field [22].

All of the above-mentioned properties concern the use of monomeric metal complexes, i.e., containing a single metal per molecule. However, gathering several metal complexes in a single and larger entity may offer enhanced properties. Indeed, it has been demonstrated that nanoscale entities may induce the EPR (enhanced permeability and retention) effect, which enables targeted drug delivery to a solid tumor, even if this effect is largely dependent on the patient’s pathological and physiological conditions [23]. A critical review of this effect has been published recently [24]. Among the nanometric objects suitable to observe the EPR effect are dendrimers [25], outstanding candidates for improved therapy and diagnosis [26]. Dendrimers [27] are hyperbranched macromolecules, generally constituted of repetitive monomers, such as polymers, but synthesized step-by-step instead of a single step in polymerization reactions [28]. Dendrimers possess a wide range of properties and are used in various fields, such as sensing, catalysis, electronics, photonics, and nanomedicine, to name a few [29]. Different types of dendrimers are known, the most widely used being the PAMAM (poly(amidoamine)) dendrimers, having a nitrogen atom at each branching point [30]. Of interest are also more “inorganic” dendrimers, in particular, those having either phosphorus or silicon at each branching point, which were reviewed early [31] and more recently [32].

In this review, we present an overview of the use of a particular Schiff base, namely pyridine–imine, linked to the surface of dendrimers, which is suitable for complexing copper. The use of such dendrimer complexes in biology, particularly against cancers, will be the main topic of this review, which will be organized depending on the type of dendrimer. The different cell lines used to test the efficiency of dendrimers are gathered in Table 1, organized in alphabetical order, together with the corresponding references.

## 2. PAMAM Dendrimer Cu Complexes

**Figure 1:**
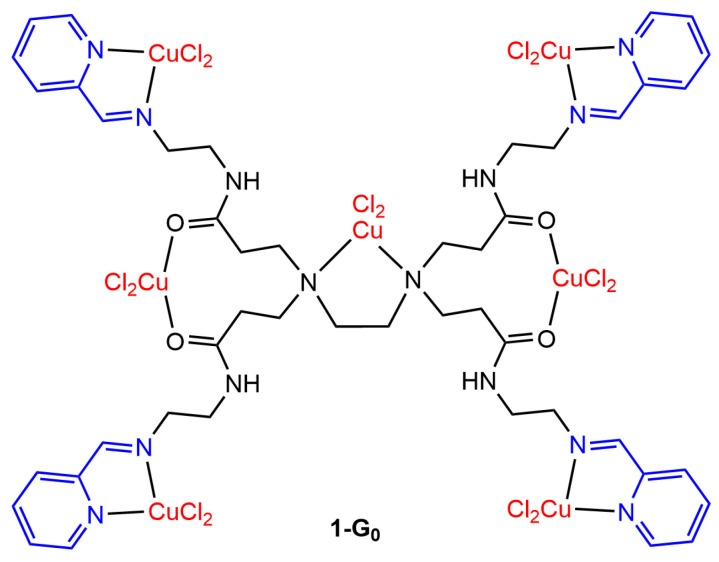
Copper complex of the generation 0 PAMAM dendrimer functionalized with four pyridine–imine groups.

Despite the fact that the PAMAM dendrimers are the most popular, they have been scarcely functionalized with pyridine–imine complexes, and only one biological evaluation has been published to the best of our knowledge. A small PAMAM dendrimer (generation 0) functionalized with four pyridine–imine ligands was used for the complexation of CuCl_2_. In fact, not only four but seven CuCl_2_ were complexed by this small dendrimer, as the C=O amide groups and the tertiary amines in the internal structure were also able to complex CuCl_2_ (Figure 1). This compound, **1-G_0,_** was found to be more toxic than cisplatin against leukemia cells (MOLT-4) and breast cancer cells (MCF-7, cisplatin-resistant cell line) but is also very toxic against the benign Chang Liver cells [34]. 

## 3. Phosphorus Dendrimers and Dendrons

### 3.1. Phosphorus Dendrimers

**Figure 2:**
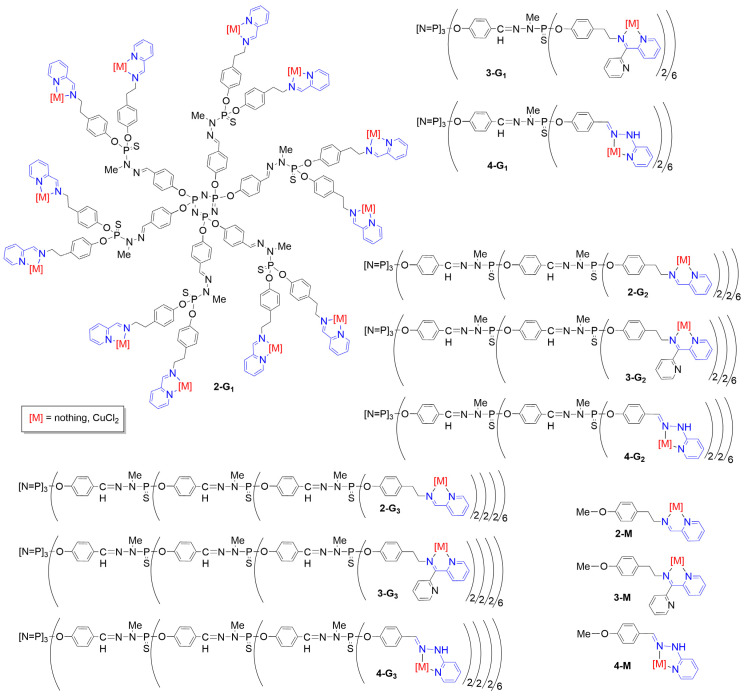
Phosphorus dendrimers from generations 1 to 3 functionalized with three types of pyridine–imine ligands and complexes and the corresponding monomers. Only the full structure of the first generation functionalized with *N*-(pyridin-2-ylmethylene) ethanamine ligand is shown. All the other dendrimers are represented in a linear form, with parenthesis after each branching point.

Phosphorus dendrimers, essentially of type poly(phosphorhydrazone) (PPH), possess a thiophosphate derivative at each branching point [50,51]. The first example of this type of dendrimers functionalized with pyridine–imine ligands for the complexation of copper was not designed for biological assays but for catalysis [52]. The very first example of PPH dendrimers complexing copper through pyridine–imine/hydrazone ligands and used for biological purposes concerned a series of nine dendrimers, from generation 1 (12 pyridine–imine) to generation 3 (48 pyridine–imine), functionalized with three different types of pyridine–imine groups, namely *N*-(pyridin-2-ylmethylene) ethanamine (**2-Gn**, n = 1–3), *N*-(di(pyridin-2-yl)methylene) ethanamine (**3-Gn**, n = 1–3), and 2-(2-methylenehydrazinyl) pyridine (**4-Gn**, n = 1–3), as shown in Figure 2. The corresponding monomers (**x-M**, with x = 2–4) were also synthesized [35]. 

**Figure 3:**
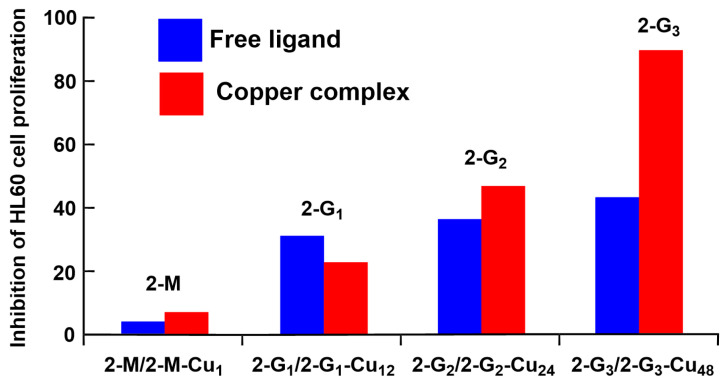
Percentage of inhibition of the proliferation of the HL60 cell line at 1 µM of all compounds from series **2-**compounds.

Both the non-complexed and complexed dendrimers and the corresponding monomers were tested for their cytotoxicity activity. The cell proliferation inhibitory effects of the series **2**-compounds (both free and complexed) were assessed against the leukemia HL60 cell line at the 1 μM concentration, corresponding to 29 μg Cu/**2-M**, 348 μg Cu/**2-G_1_**, 696 μg Cu/**2-G_2_**, and 1392 μg Cu/**2-G_3_**. The third-generation dendrimer complexing copper (**2-G_3_-Cu_48_**) was the most efficient when considering the number of moles (Figure 3), but when considering the quantity of copper, the monomer was the most efficient. However, no experiment was carried out with an identical quantity of copper in all cases [35].

**Figure 4:**
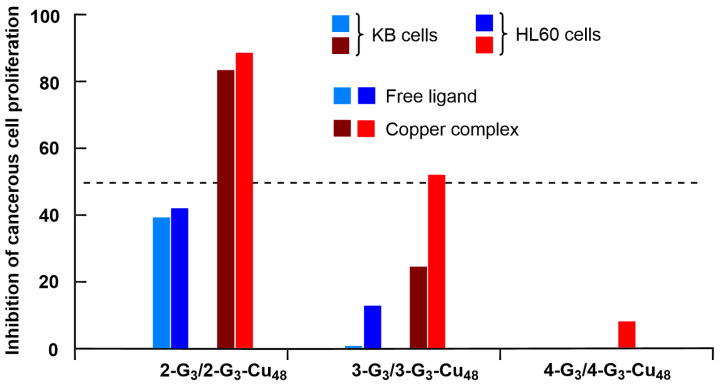
Percentage of inhibition at 1 µM of the proliferation of the KB and HL60 cell lines at 1 µM of the third-generation dendrimers with either free or complexing CuCl_2_.

In the following experiment, the influence of the type of ligand on the efficiency was tested on the HL60 cell line and the KB cell line (epidermal carcinoma). As the maximum inhibition was observed with the third generations, **2-G_3_** and **2-G_3_-Cu_48_** (Figure 3), only the third generations of the other dendrimers were tested in this case (Figure 4). In all cases, the copper complexes at 1 µM were more active than the non-complexed dendrimers at the same concentration, but the type of ligand played a crucial role. The *N*-(pyridin-2-ylmethylene) ethanamine ligand (series **2**-) was very active, whereas the *N*-(di(pyridin-2-yl)methylene) ethanamine (series **3**-) was poorly active, and 2-(2-methylenehydrazinyl) pyridine (series **4**-) had practically no activity [35].

**Figure 5:**
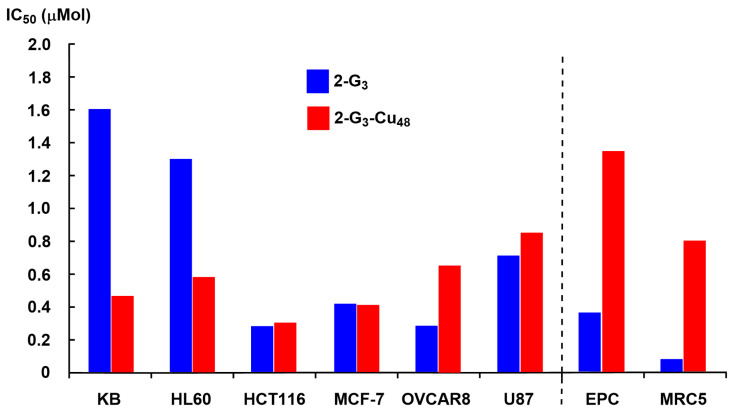
IC_50_ of free (**2-G_3_**) and complexed dendrimer (**2-G_3_-Cu_48_**) in six cancerous cell lines (left part) and two non-cancerous cell lines (right part).

Finally, only the most active dendrimers (**2-G_3_** and **2-G_3_-Cu_48_**) were tested on a panel of cancerous and non-cancerous cell lines. In this case, the IC_50_ was measured. It indicates the concentration of a substance that is necessary to kill 50% of the cells and, therefore, the potency of the compound. It is important to develop compounds with a good selectivity towards cancer cells, compared to normal ones, in order to obtain promising cytotoxic agents. Experiments were carried out on the HCT116 (human colon cancer), MCF-7 (hormone-responsive human breast cancer), OVCAR8 (ovarian carcinoma), and U87 (human glioblastoma-astrocytoma, epithelial-like) cancerous cell lines, and with the non-cancerous cell lines MRC5 (proliferative human lung fibroblasts) and the quiescent EPC (endothelial progenitor cells, *Cyprinus carpio*). The results are shown in Figure 5. Interestingly, the copper complex **2-G_3_-Cu_48_** is more toxic towards the cancerous cells than towards the non-cancerous cells, contrarily to the non-complexed dendrimer **2-G_3_** [35].

The same dendrimer complexes (**2-Gn-Cu_x_**, **3-Gn-Cu_x_**, and **4-Gn-Cu_x_**, with *n* = 1–3 and x = 12 to 48) and monomer complexes (**2-M-Cu_1_**, **3-M-Cu_1_**, and **4-M-Cu_1_**) were then studied by EPR (electron paramagnetic resonance) in the absence and presence of normal (MRC5) and cancer (HCT116) cells. However, in those cases, CuCl_2_ is added to the free dendrimers and monomers in solution, i.e., it is not the preformed complex that is studied, contrarily to the previous experiments. In the absence of cells, the experiments were carried out in DMF. It was shown that in series **2**-, a chelate complex was formed (two pyridine–imines for one Cu^II^), stabilizing the complex, which is more difficult to form in the case of the other series. In all cases, a better complexation was observed with the highest generations (**G_3_**) due to the increased density of binding sites. An opalescence in the solutions of series **4**- indicates a phase separation and the formation of a precipitate, emphasizing the weak complexation of Cu^II^, correlating with the very low efficiency of this series in the biological evaluation (Figure 4). For the weak complexes, the coordination sphere of copper was completed with oxygen atoms from the solvent. In view of both the EPR results in solvent and the biological properties, only the EPR properties of the Cu complex of **2-G_3_** were studied in solution and in the presence of cells. It was shown, in particular, that the EPR signal is more intense in the presence of the HCT116 cancerous cells than in the presence of the MRC5 non-cancerous cells. Such behavior is correlated with a stronger binding of Cu-**2-G_3_** with HCT116 cells compared to the MRC5 cells [39].

Additional biological experiments were carried out to determine the mechanism of action of both dendrimers **2-G_3_** and **2-G_3_-Cu_48_**. It was shown that both dendrimers penetrated inside cells by endocytosis but behaved differently inside the cells. Dendrimer **2-G_3_** moderately activated caspase-3, moderately initiating the apoptotic cascade leading to cell death. On the contrary, dendrimer **2-G_3_-Cu_48_** strikingly reduced the caspase-3 content and activity but promoted the translocation of the Bax protein to the mitochondrial compartment, resulting in the release of apoptosis-inducing factor. Such a release induced the activation of the caspase-independent apoptotic pathway through severe DNA fragmentation without alteration of the cell cycle. These different behaviors correlate with the weak anti-proliferative efficiency observed with **2-G_3_** and the better efficiency of **2-G_3_-Cu_48_**, which possesses a distinctive mode of action [38].

**Figure 6:**
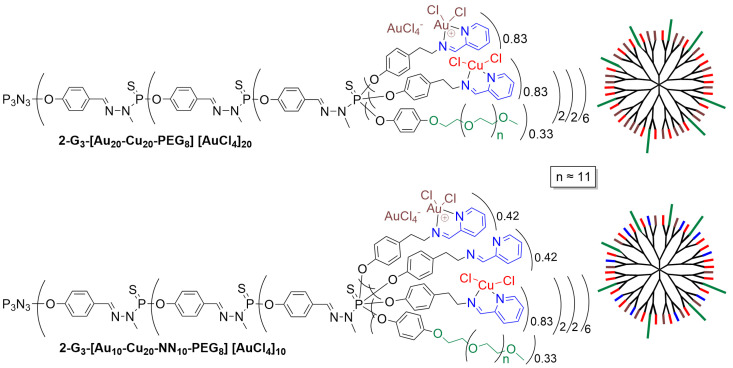
Random functionalization on the surface of the third-generation dendrimers, with polyethylene glycol (PEG) moieties, copper and gold complexes, and eventually free pyridine–imine ligands.

In a further step of this project, compound **2-G_3_** and analogs, bearing a few PEG (polyethylene glycol) moieties to increase the solubility in water, were used for complexing mixtures of copper and gold. Contrarily to copper, it is not AuCl_3_ that is complexed by the pyridine–imine ligand, but [AuCl_2_]^+^ with [AuCl_4_]^-^ as the counter ion. Two examples of this stochastic (random) functionalization on the surface of the third-generation dendrimer are shown in Figure 6 [36]. 

**Figure 7:**
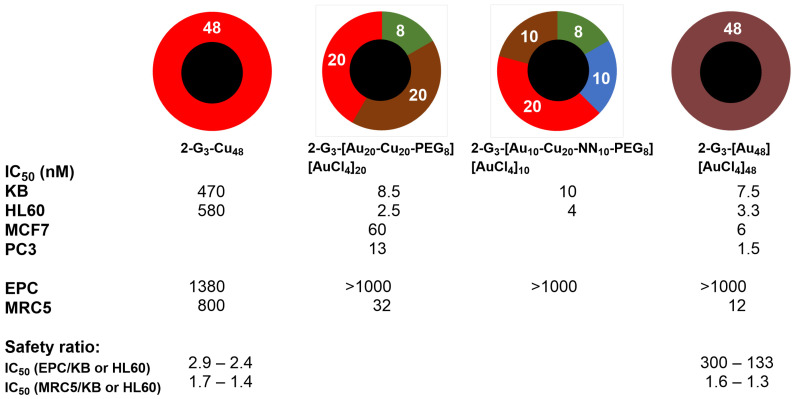
IC_50_ values of four dendrimeric complexes functionalized with copper (red), gold (brown), or a mixture of both metals and free pyridine–imine (blue) or PEG (green) toward cancerous cells (KB and HL60) and non-cancerous cells (EPC and MRC5).

The biological properties of both randomly functionalized dendrimers shown in Figure 6 were tested in comparison with the dendrimers fully complexed with either copper (**2-G_3_-Cu_48_**) or gold (**2-G_3_-[Au_48_][AuCl_4_]_48_**), as shown in Figure 7. The dendrimeric complexes fully functionalized with gold metal are far more efficient than the corresponding copper complex toward cancerous cells (KB and HL60), with IC_50_ in the nanomolar range for the gold complex compared to the hundreds nanomolar range for the copper complex. Both dendrimers were tested against the non-cancerous cells EPC and MRC5. Both compounds displayed relatively low toxicity towards EPC cells, but deceptively, the gold complexes are highly toxic toward the MRC5 cells. Thus, there is a very good safety index for the gold complex, but only for EPC cells [36]. 

In view of the large difference observed between copper and gold, the question of the quantity of gold necessary to obtain the same effect arose. Dendrimer **2-G_3_-[Au_20_-Cu_20_-PEG_8_][AuCl_4_]_20_**, possessing the same quantity of gold and copper, plus 8 PEGs, was highly toxic towards both KB and HL60 cancerous cells and poorly toxic towards the non-cancerous EPC cells (see Figure 7). Even dendrimer **2-G_3_-[Au_10_-Cu_20_-NN_10_-PEG_8_][AuCl_4_]_10_**, having only 10 **[AuCl_2_][AuCl_4_]** complexes, had exactly the same properties. Both dendrimers **2-G_3_-[Au_20_-Cu_20_-PEG_8_][AuCl_4_]_20_** and **2-G_3_-[Au_48_][AuCl_4_]_48_** were then tested towards two other cancerous cell lines, MCF-7 (human breast adenocarcinoma cell line) and PC3 (prostatic small cell carcinoma). The gold complex was more efficient than the copper-gold complex against both cell lines (Figure 7) [36].

In another experiment, the copper complex **2-G_3_-Cu_48_** was used in combination with several anticancer agents with different modes of action: camptothecin, cisplatin, paclitaxel, doxorubicin, and MG132. These combinations were tested against KB and HL60 cell lines at the active dose of each compound to detect the inhibition of cell proliferation. No effect was observed with cisplatin towards KB cells, but an increased efficiency was observed against the HL60 cell line. An additive effect was observed with the combinations of **2-G_3_-Cu_48_** with paclitaxel or MG132 (proteasome inhibitor). More interestingly, a synergistic effect was observed in the combination of **2-G_3_-Cu_48_** with doxorubicin, ongoing from ca. 30% inhibition with doxorubicin alone to ca. 90% with doxorubicin + **2-G_3_-Cu_48_** [45].

The minimal inhibitory concentrations (MIC) and the minimal bactericidal concentrations (MBC) of some of these copper and gold complexes of dendrimers against numerous bacteria and yeast were also measured. It was shown that dendrimer **2-G_3_-[Au_48_][AuCl_4_]_48_** had the highest antimicrobial activity, whereas dendrimer **2-G_3_-[Au_10_-Cu_20_-NN_10_-PEG_8_][AuCl_4_]_10_** had the highest antifungal activity. Interestingly, and contrarily to the results in oncology shown above, a marked synergistic effect was found for the antifungal activity when both copper and gold were present together in the same dendrimer [36]. 

### 3.2. Phosphorus Dendrons

**Figure 8:**
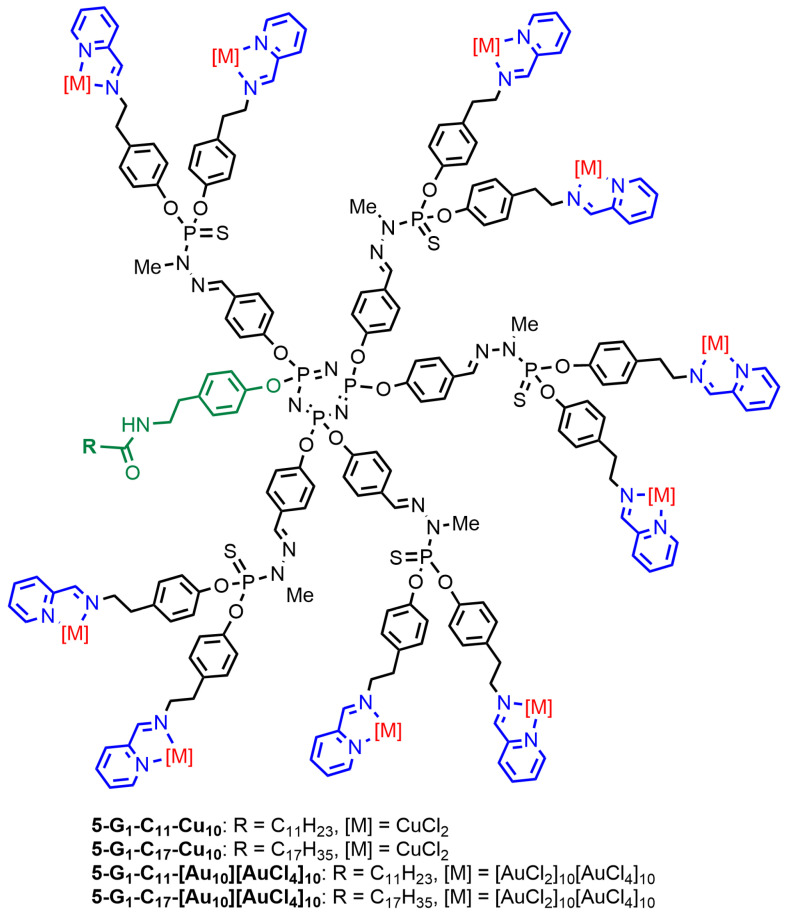
First-generation dendrons bearing a C_11_ or C_17_ alkyl chain at the core and complexing either copper or gold on the surface.

A series of phosphorus dendrons was synthesized thanks to the versatile reactivity of the cyclotriphosphazene core and, in particular, the possibility to differentiate one function among six to produce AB_5_ derivatives [53]. The terminal functions of these dendrons are identical to those of compound **2-Gn,** shown in Figure 2. The function at the core is an alkyl chain incorporating either 11 or 17 carbon atoms linked to the dendrons through a phenol amide. Only the first generations of these dendrons (**5-G_1_-C_11_**, **5-G_1_-C_17_**) were synthesized, and they were used for complexing either copper or gold (Figure 8). These dendrons self-associate in water to form aggregates whose hydrodynamic size measured by dynamic light scattering (DLS) depends mainly on the length of the alkyl chain, from ca. 260 nm for the dendrons equipped with the C_11_ chain to ca. 500 nm with the C_17_ chain [47]. 

**Figure 9:**
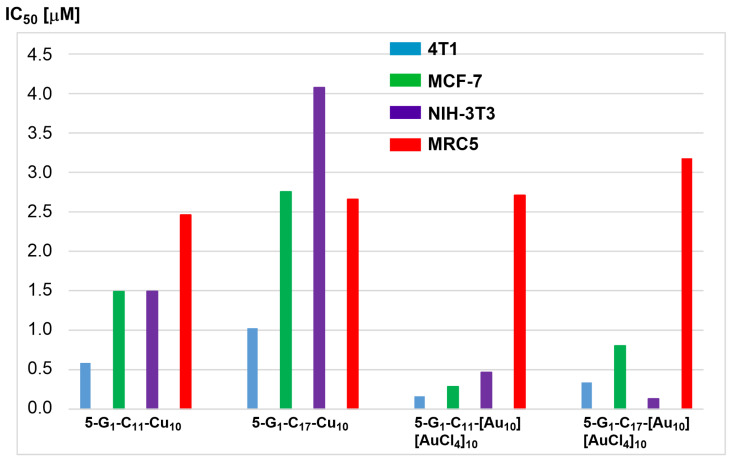
IC_50_ values of the dendrons shown in Figure 8 towards cancerous (4T1 and MCF-7) and non-cancerous (NIH-3T3 and MRC5) cell lines.

The efficiency of all these dendrons was then tested against two different breast cancer cell lines, 4T1 (mouse breast adenocarcinoma cells) and MCF-7 (human breast cancer), and normal cells, NIH-3T3 (normal fibroblast) and MRC5. The corresponding metal-free dendrons were tested but displayed no anti-proliferative activity (IC_50_ values >100 µm). The four dendron complexes were then tested against the four cell lines, as shown in Figure 9. The gold complexes **5-G_1_-C_11_-[Au_10_][AuCl_4_]_10_** and **5-G_1_-C_17_-[Au_10_][AuCl_4_]_10_** are more efficient against both cancerous cell lines than the copper complexes **5-G_1_-C_11_-Cu_10_** and **5-G_1_-C_17_-Cu_10_**. However, the gold complexes are highly toxic toward the non-cancerous cells NIH-3T3, whereas the copper complexes are less toxic (Figure 9). All dendrons shown in Figure 8 are less toxic against the non-cancerous cells MRC5. The length of the alkyl chain has a larger influence in the case of the copper complexes than in the case of the gold complexes. The longer chain decreases the efficiency of dendron **5-G_1_-C_17_-Cu_10_** [47].

**Figure 10:**
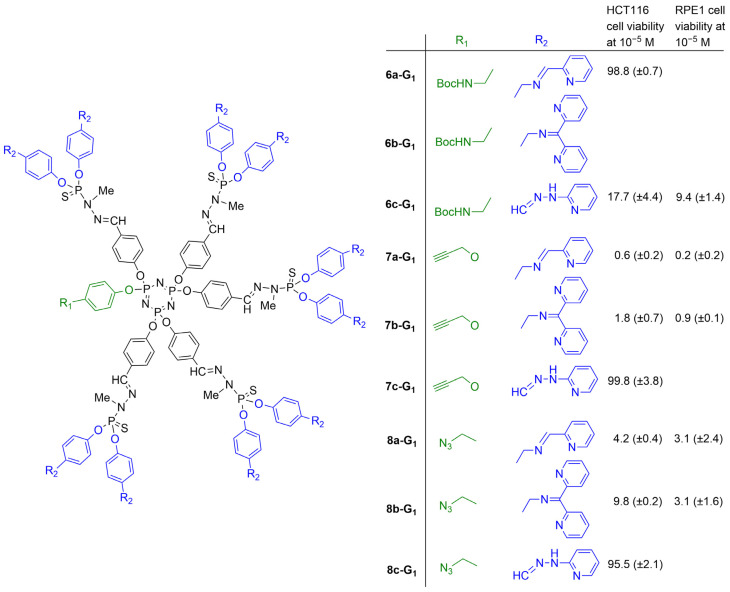
Three series of dendrons bearing diverse types of pyridine–imine/hydrazone derivatives on the surface and a reactive group at the core. Evaluation of their toxicity at 10^−5^ M.

Several other series of phosphorus dendrons with diverse functions at the core and pyridyl–imine/hydrazone derivatives as terminal functions (Figure 2, but without metal) were synthesized (Figure 10). The functions at the core were chosen for their ability to react with functionalized graphene oxide (GO). Dendrons having a Boc-protected amine at the core (dendrons **6a,b,c-G_1_**) were tentatively deprotected to generate a primary amine suitable to react with GO modified with acyl chlorides. Unfortunately, the trifluoroacetic acid used for the deprotection of the amine at the core induced a partial cleavage of the imine bonds on the surface of the dendrons and precluded the use of these dendrons for grafting to GO. However, the biological properties of the Boc-protected dendrons were tested against the HCT116 cells. Only dendrimer **6c-G_1_** was relatively active at a concentration of 10^−5^ M, but it was more toxic against the non-cancerous cells RPE1 (Figure 10) [40].

**Figure 11:**
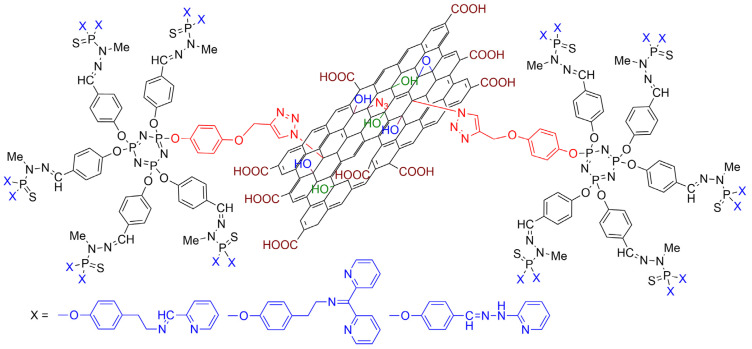
Illustration of the grafting of dendrons onto graphene oxide (GO) by click chemistry.

Two other series of dendrons were synthesized, bearing either an alkyne or an azide at the core. Both series were used in “click” chemistry [54,55] with GO functionalized with either azide or alkyne. The result of the reaction of the dendrons bearing an alkyne at the core (dendrons **7a,b,c-G_1_**, Figure 10) with graphene oxide functionalized with azides is shown in Figure 11. The biological properties of the dendrons alone and of the dendrons grafted to GO were tested towards the cancerous cell line HCT116 and the non-cancerous cells of human retinal pigment epithelial-1 (RPE1). Four dendrons (**7a-G_1_**, **7b-G_1_**, **8a-G_1_**, and **8b-G_1_**) were active against the HCT116 cell line at a concentration of 10^−5^ M but not at 10^−6^ M. These four dendrons were then tested with the non-cancerous RPE1 cells and were deceptively found to be even more toxic, as shown in Figure 10. The GO functionalized by the six dendrons was also tested against the HCT116 cell line, but none of them were really active [41].

**Figure 12:**
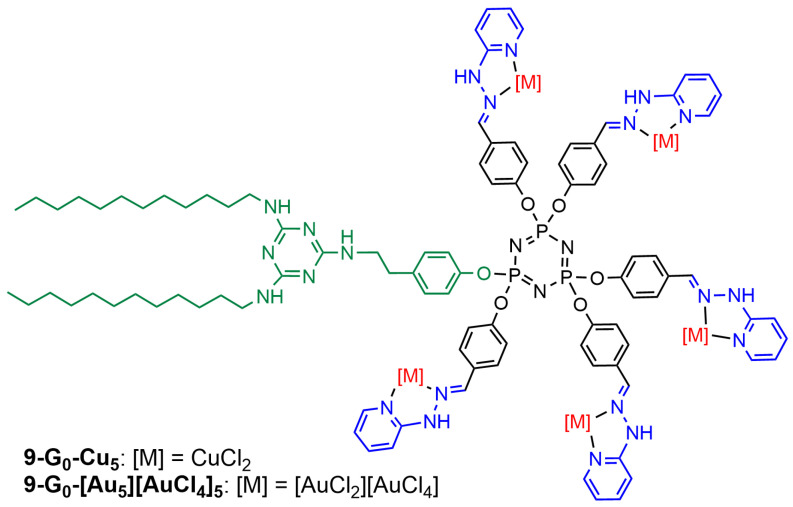
Generation zero dendrons bearing two alkyl chains at the core and five copper or gold complexes on the surface.

**Figure 13:**
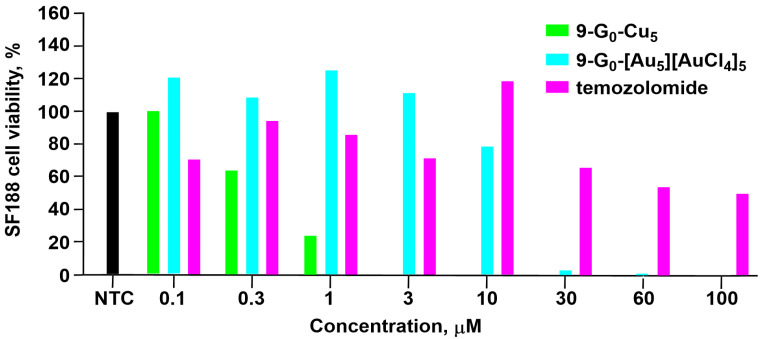
Comparison of viability of SF188 cells in the presence of either **9-G_0_-Cu_5_** or **9-G_0_-[Au_5_][AuCl_4_]_5_** compared to temozolomide, the clinical standard. NTC, non-treated control.

The last example of phosphorus dendrons functionalized with pyridine–imine groups concerns generation zero. It has a triazine ring bearing two nitrogen C_12_ alkyl chains and is linked to the core by the third nitrogen via a *para*-ethylphenoxy fragment. It also integrates in its end five pyridyl hydrazones capable of complexing either copper (**9-G_0_-Cu_5_**) or gold (**9-G_0_-[Au_5_][AuCl_4_]_5_**), as shown in Figure 12. These dendrons form single micelles (diameter ca. 9 nm) with **9-G_0_-Cu_5_** and multi-micellar aggregates (diameter ca. 60 nm) with **9-G_0_-[Au_5_][AuCl_4_]_5_** in water, as shown both by DLS and TEM images. Both dendrons were tested against three lines of glioblastoma stem cells (BTSC233, JHH520, and NCH644), pediatric glioma cells SF188, and two growth variants of the U87 glioblastoma cells (U87a and U87s). In all experiments, a comparison with temozolomide (IC_50_ > 100 µM), the clinical standard of care for glioblastoma, is made. In most cases, the copper complex is more efficient than temozolomide, whereas the gold complex is only slightly more efficient than temozolomide at high concentrations. At the concentration of 3 µM, all SF188 cells were killed by **9-G_0_-Cu_5_**, whereas no SF188 cells were killed by **9-G_0_-[Au_5_][AuCl_4_]_5_**, and only about 30% of the SF188 cells were killed by temozolomide. At the concentration of 100 µM, all the SF188 cells were killed by **9-G_0_-Cu_5_** and by **9-G_0_-[Au_5_][AuCl_4_]_5_**, but only about 50% of the SF188 cells were killed by temozolomide. Figure 13 displays the results obtained against the SF188 cell line [33].

## 4. Carbosilane Dendrimers

**Figure 14:**
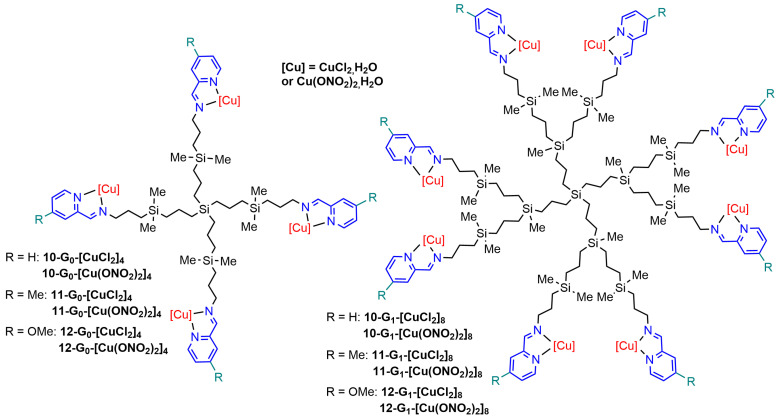
Generations zero and one carbosilane dendrimers functionalized with copper complexes of pyridine–imine.

Carbosilane dendrimers are constituted of short alkyl chains linked through silicon at the branching points [56]. Generations zero and one carbosilane dendrimers were functionalized with four and eight pyridine–imine derivatives, respectively, which were used for complexing copper (Figure 14).

### 4.1. Anticancer Properties of Carbosilane Dendrimers

The anticancer properties of carbosilane dendrimers were, in particular, studied, as was done previously with the phosphorus dendrimers. The first paper about copper complexes of carbosilane dendrimers described the synthesis of dendrimers **10-G_0_-[CuCl_2_]_4_** and **10-G_1_-[CuCl_2_]_8_**, and their efficiency against two cancerous cell lines, PC3 (human prostate) and HeLa (human cervical). IC_50_ values were found in the μM range, with generation 0 (IC_50_ 4.5 μM) being more efficient against the PC3 cell line than generation 1 (IC_50_ 12.5 μM). Both generations have the same efficiency against the HeLa cell line, with IC_50_ at ca. 10 μM [42].

**Figure 15:**
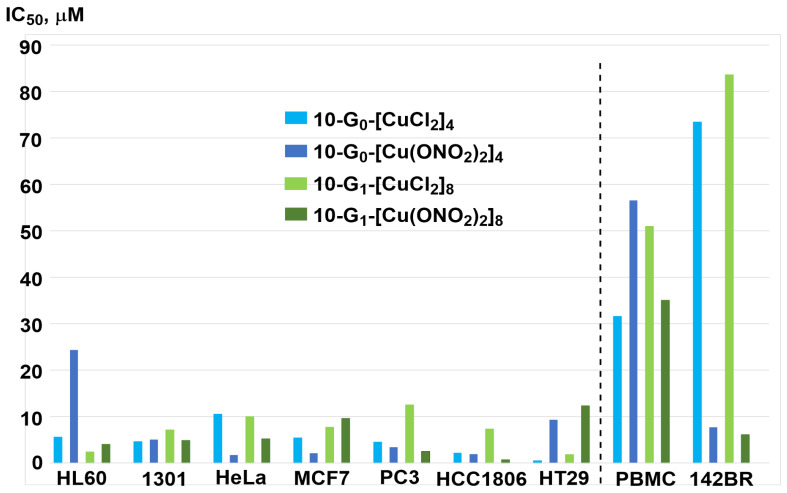
IC_50_ values of carbosilane dendrimers towards cancerous cell lines (left) and healthy cell lines (right).

Dendrimers **10-G_0_-[Cu(ONO_2_)_2_]_4_** and **10-G_1_-[Cu(ONO_2_)_2_]_8_** were also synthesized, and the hemotoxicity of the four dendrimers of series **10** was measured by the intensity of membrane destruction on blood from healthy donors. Dendrimers complexing Cu(NO_3_)_2_ are more toxic than those complexing CuCl_2_. The four dendrimers of the series **10-Gn-[Cu]** were then tested against two cancerous cell lines of leukemia (HL60 and 1301) and against the normal cell line PBMC (peripheral blood mononuclear cells). Figure 15 displays the IC_50_ values. Interestingly, all dendrimers were more toxic against both cancerous cell lines than towards the PBMCs [46]. 

The behavior of the same dendrimers of series **10-Gn-[Cu]** towards cell membrane models (cethyl-trimethylammonium bromide (CTAB) micelles and lecithin liposomes) was investigated by EPR (electron paramagnetic resonance). The results indicated that generation 1 **10-G_1_-[CuCl_2_]_8_** dendrimers interact more strongly with the model membranes than other dendrimers. The dendrimers were tested against other cancerous cell lines HeLa, MCF-7, and HCC1806 (normal and resistant breast cancer cells), PC3, and HT29 (colorectal tumor), and one healthy cell line 142BR (human fibroblasts). Figure 15 also displays these additional results, with some differences compared to the previous results. In particular, both dendrimers bearing Cu(NO_3_)_2_ complexes **10-G_0_-[Cu(ONO_2_)_2_]_4_** and **10-G_1_-[Cu(ONO_2_)_2_]_8_** are highly toxic towards the healthy cell line 142BR. Dendrimer **10-G_0_-[Cu(ONO_2_)_2_]_4_** is both the most efficient against the resistant prostate cancer cell line PC3 and the easiest to synthesize; it was also tested in *ex vivo* experiments with mice bearing xenografted human prostate cancer. Up to 37% smaller tumor sizes were observed in the mice treated with **10-G_0_-[Cu(ONO_2_)_2_]_4_** compared to untreated mice [37]. Additional experiments were carried out against the myeloid U937 tumor cells [49].

The four dendrimers of series **10-Gn-[Cu]** were also tested in combination with pro-apoptotic siRNAs against breast cancers, which may result in the induction of apoptosis. Associations of dendrimers with siRNAs formed dendriplexes in the ratio 1:25–1:50 (siRNA/dendrimer) for the CuCl_2_ complexes and 1:15–1:25 for the Cu(NO_3_)_2_ complexes; thus, the chosen molar ratio for all compounds was **10-Gn-[Cu]**/siRNA = 30. The size of the dendriplexes was measured by DLS, affording sizes ranging from 980 to 1110 nm with generations zero and 290–340 nm with the first generations. The uptake of the dendriplexes in MCF-7 cells was more efficient with the first generation than with the zero-generation dendrimers. The dendriplexes obtained with dendrimer **10-G_1_-[Cu(ONO_2_)_2_]_8_** are the most efficiently internalized. The viability of MCF-7 cells after 72 h of incubation is largely affected by the presence of the dendriplexes, and the best results were obtained with dendriplexes formed with the first generations [48]. The dendriplexes were also shown to be more active against MCF-7 cells than the dendrimers alone.

**Figure 16:**
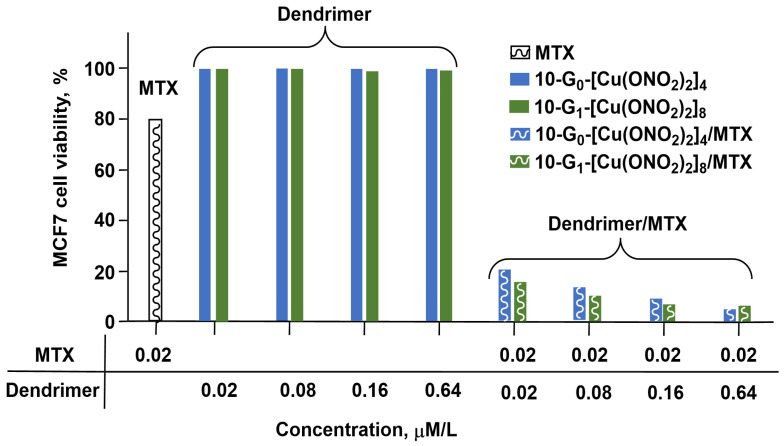
MCF-7 cell viability in the presence of methotrexate (MTX) alone, dendrimers alone, or combination of methotrexate and dendrimers.

In another type of association, the same dendrimers were used in combination with conventional antitumor drugs, doxorubicin, methotrexate, and 5-fluorouracil. These combinations were tested against two cancerous cell lines, MCF-7 and HepG2 (human liver carcinoma). The efficiency of the conventional drugs significantly increased when associated with the dendrimers. This association resulted in an increase in the reactive oxygen species (ROS) levels and in the depolarization of mitochondrial membranes. The presence of copper ions enhanced the anticancer properties of the whole system and induced both apoptosis and necrosis. Figure 16 displays the most spectacular example of the efficiency of these combinations on the MCF-7 cell viability using methotrexate (MTX) as the conventional drug at a concentration in which alone the cell viability decreased only to 80% (0.02 μM/L), and the dendrimers **10-G_0_-[Cu(ONO_2_)_2_]_4_** and **10-G_1_-[Cu(ONO_2_)_2_]_8_** at different concentrations [44].

**Figure 17:**
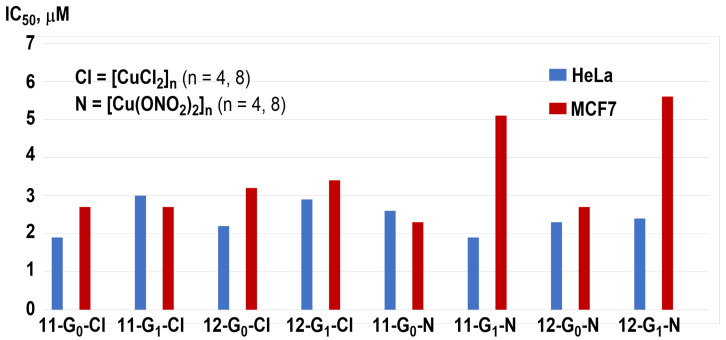
IC_50_ of the functionalized carbosilane dendrimer complexes against HeLa and MCF-7 cell lines.

In order to perform a larger structure/activity study, pyridine–imine derivatives functionalized in the *para* position (relative to the nitrogen atom of pyridine) were also synthesized (series **11-Gn-[Cu]** and **12-Gn-[Cu]** in Figure 14). The IC_50_ values of all these dendrimers were measured against two cancerous cell lines, HeLa and MCF-7 (Figure 17). All dendrimers display potent antitumor activity in the micromolar range, lower than those observed with the non-substituted derivatives. No clear trends depending on the nature of the pyridine–imine substituent could be deduced from these results. As generation zero seems more potent than generation-one dendrimers, an in-depth biological evaluation was carried out with these small dendrimers. Dendrimers **11-G_0_-[CuCl_2_]_4_** and **12-G_0_-[Cu(ONO_2_)_2_]_4_** strongly affected U937 tumor myeloid cells viability, by inducing late apoptotic/necrotic cells in 75% and 95%, respectively [43], while they poorly affected the viability of PBMC normal cells.

### 4.2. Antibacterial Properties of Carbosilane Dendrimers

Besides the anticancer properties, the four carbosilane dendrimers of series **10-Gn-[Cu]** (R = H in Figure 14) were also tested against Gram-positive (*Staphylococcus aureus*) and Gram-negative (*Escherichia coli*) bacteria, and biofilms of *Staphylococcus aureus*. Generations zero (**10-G_0_-[CuCl_2_]_4_** and **10-G_0_-[Cu(ONO_2_)_2_]_4_**) are more efficient than generation one. They display MIC values in the range of 2–4 mg/L against *S. aureus*, 4–8 mg/L against *E. coli*, and MIB values in the range of 4–8 mg/L against both bacteria. Both generation-zero dendrimers were also tested for preventing *S. aureus* biofilm formation. Both compounds had an MIC of 8 mg/L and an MIB in the range of 4–8 mg/L for preventing biofilm formation [57]. 

The functionalized dendrimers (generations zero of series **11-G_0_-[Cu]** (R = Me) and **12-G_0_-[Cu]** (R = OMe)) were also tested against *Staphylococcus aureus* biofilms, as were the unfunctionalized dendrimers (**10-Gn-[Cu]** (R = H)). All compounds were active but less active than the corresponding unfunctionalized dendrimers **10-Gn-[Cu] [58]**.

## 5. Conclusions

We have shown in this review that pyridine–imine copper complexes of dendrimers offer interesting anticancer properties *in vitro* and, in some cases, *in vivo* (mice). The large panel of cancerous cells was studied, in most cases, only one or two times, and thus precludes a real SAR (structure–activity relationship) study. The only exception concerns the MCF-7 cisplatin-resistant breast cancer cells, which have been studied in eight publications (Table 1). All tested dendrimers and dendrons against MCF-7 cells display IC_50_ activities in the μMolar range. The only exception concerns the randomly functionalized phosphorus dendrimer **2-G_3_-[Au_20_-Cu_20_-PEG_8_][AuCl_4_]_20_**, which displays the IC_50_ at a very low value of 13 ± 2 nM. However, this low value is essentially due to the presence of gold, in addition to copper [36]. 

It should be noted that pyridine–imine copper complexes are found essentially on the surface of only two types of dendrimers, namely phosphorhydrazone and carbosilane dendrimers. This fact emphasizes that main group element dendrimers have many interesting properties, even if they are frequently unrecognized. Furthermore, we hope that this review will also convince researchers working with other types of dendrimers of the usefulness of this type of ligand and complex for other biological experiments. According to literature reviews, the current treatments for cancers [59], and even those foreseen [60], do not display any information about the use of dendrimers in clinic. It means that additional work is really needed in the field of dendrimers to bring them to clinical trials [61]. 

## Figures and Tables

**Table 1:** Cell names, cell lines, and references.

Cell Names	Cell Lines ^1^	References
BTSC233	Glioblastoma stem cells	[33]
Chang liver	Liver cells, *non-cancerous*	[34]
EPC	Endothelial progenitor, *Cyprinus carpio*, *non-cancerous*	[35,36]
HCC1806	Resistant breast cancer cells	[37]
HCT15	Colon cancer	[38]
HCT116	Colon cancer	[35,38,39,40,41]
HeLa	Cervical cancer cells	[37,42,43]
HepG2	Liver carcinoma	[44]
HL60	Leukemia	[35,36,38,45,46]
HT29	Colorectal tumor	[37]
JHH520	Glioblastoma stem cells	[33]
KB	Epidermal carcinoma	[35,36,45]
MCF-7	Breast cancer cells, cisplatin resistant	[34,35,36,37,38,43,44,47,48]
MOLT-4	Leukemia	[34]
MRC5	Proliferative lung fibroblasts, *non-cancerous*	[35,36,39,47]
NCH644	Glioblastoma stem cells	[33]
NIH-3T3	Normal fibroblasts, *non-cancerous*	[47]
OVCAR8	Ovarian carcinoma	[35]
PBMC	Peripheral blood mononuclear cells, *non-cancerous*	[43,46]
PC3	Prostatic small cell carcinoma	[36,37,42]
RPE1	Retinal pigment epithelial-1, *non-cancerous*	[40,41]
SF188	Pediatric glioma cells	[33]
U87	Glioblastoma-astrocytoma, epithelial-like	[33,35]
U937	Myeloid tumor cells	[43,49]
1301	Leukemia	[46]
142BR	Fibroblasts, *non-cancerous*	[37]
4T1	Mouse breast adenocarcinoma cells	[47]

^1^ of human origin, if not indicated.

## Data Availability

No new data were created or analyzed in this study. Data sharing is not applicable to this article.

## References

[1] Rosenberg B., Vancamp L., Trosko J.E., Mansour V.H. (1969). Platinum compounds: A new class of potent antitumour agents. Nature.

[2] Kelland L. (2007). The resurgence of platinum-based cancer chemotherapy. Nat. Rev. Cancer.

[3] Adhikari S., Nath P., Das A., Datta A., Baildya N., Duttaroy A.K., Pathak S. (2024). A review on metal complexes and its anti-cancer activities: Recent updates from in vivo studies. Biomed. Pharmacother..

[4] Temesgen A., Ananda Murthy H.C., Zereffa Enyew A., Revathi R., Venkatesha Perumal R. (2023). Emerging trends in metal-based anticancer agents: Drug design to clinical trials and their mechanism of action. Chem. Select.

[5] Lucaciu R.L., Hangan A.C., Sevastre B., Oprean L.S. (2022). Metallo-drugs in Cancer Therapy: Past, Present and Future. Molecules.

[6] Marzano C., Pellei M., Tisato F., Santini C. (2009). Copper complexes as anticancer agents. Anti-Cancer Agents Med. Chem..

[7] Santini C., Pellei M., Gandin V., Porchia M., Tisato F., Marzano C. (2014). Advances in copper complexes as anticancer agents. Chem. Rev..

[8] Feng Q., Huo C., Wang M., Huang H., Zheng X., Xie M. (2024). Research progress on cuproptosis in cancer. Front. Pharmacol..

[9] Chen L., Min J., Wang F. (2022). Copper homeostasis and cuproptosis in health and disease. Signal Transduct. Target. Ther..

[10] Wang Z., Jin D., Zhou S., Dong N., Ji Y., An P., Wang J., Luo Y., Luo J. (2023). Regulatory roles of copper metabolism and cuproptosis in human cancers. Front. Oncol..

[11] Xie J., Yang Y., Gao Y., He J. (2023). Cuproptosis: Mechanisms and links with cancers. Mol. Cancer.

[12] Wang Y., Chen Y., Zhang J., Yang Y., Fleishman J.S., Wang Y., Wang J., Chen J., Li Y., Wang H. (2024). Cuproptosis: A novel therapeutic target for overcoming cancer drug resistance. Drug Resist. Updates.

[13] De Luca A., Barile A., Arciello M., Rossi L. (2019). Copper homeostasis as target of both consolidated and innovative strategies of anti-tumor therapy. J. Trace Elem. Med. Biol..

[14] Lowndes S.A., Harris A.L. (2005). The role of copper in tumour angiogenesis. J. Mammary Gland. Biol. Neoplasia.

[15] Nasulewiez A., Mazur A., Opolski A. (2004). Role of copper in tumour angiogenesis–clinical implications. J. Trace Elem. Med. Biol..

[16] Yang Y., Li M., Chen G., Liu S., Guo H., Dong X., Wang K., Geng H., Jiang J., Li X. (2023). Dissecting copper biology and cancer treatment: ‘Activating Cuproptosis or suppressing Cuproplasia’. Coord. Chem. Rev..

[17] Lelievre P., Sancey L., Coll J.-L., Deniaud A., Busser B. (2020). The Multifaceted Roles of Copper in Cancer: A Trace Metal Element with Dysregulated Metabolism, but Also a Target or a Bullet for Therapy. Cancers.

[18] Merlot A.M., Kalinowski D.S., Richardson D.R. (2013). Novel Chelators for Cancer Treatment: Where Are We Now?. Antioxid. Redox Signal..

[19] Wang W., Mo W., Hang Z., Huang Y., Yi H., Sun Z., Lei A. (2023). Cuproptosis: Harnessing Transition Metal for Cancer Therapy. ACS Nano.

[20] Denoyer D., Masaldan S., La Fontaine S., Cater M.A. (2015). Targeting copper in cancer therapy: ‘*Copper That Cancer*’. Metallomics.

[21] Richa, Kumar V., Kataria R. (2024). Phenanthroline and Schiff Base associated Cu(II)-coordinated compounds containing N, O as donor atoms for potent anticancer activity. J. Inorg. Biochem..

[22] More M.S., Joshi P.G., Mishra Y.K., Khanna P.K. (2019). Metal complexes driven from Schiff bases and semicarbazones for biomedical and allied applications: A review. Mater. Today Chem..

[23] Maeda H. (2015). Toward a full understanding of the EPR effect in primary and metastatic tumors as well as issues related to its heterogeneity. Adv. Drug Deliv. Rev..

[24] Sun R., Xiang J., Zhou Q., Piao Y., Tang J., Shao S., Zhou Z., Bae Y.H., Shen Y. (2022). The tumor EPR effect for cancer drug delivery: Current status, limitations, and alternatives. Adv. Drug Deliv. Rev..

[25] Svenson S. (2009). Dendrimers as versatile platform in drug delivery applications. Eur. J. Pharm. Biopharm..

[26] Cheng Y., Zhao L., Li Y., Xu T. (2011). Design of biocompatible dendrimers for cancer diagnosis and therapy: Current status and future perspectives. Chem. Soc. Rev..

[27] Tomalia D.A., Naylor A.M., Goddard W.A. (1990). Starburst dendrimers–Molecular level control of size, shape, surface chemistry, topology, and flexibility from atoms to macroscopic matter. Angew. Chem.-Int. Edit. Engl..

[28] Caminade A.M., Turrin C.O., Laurent R., Ouali A., Delavaux-Nicot B. (2011). Dendrimers: Towards Catalytic, Material and Biomedical Uses.

[29] Astruc D., Boisselier E., Ornelas C. (2010). Dendrimers Designed for Functions: From Physical, Photophysical, and Supramolecular Properties to Applications in Sensing, Catalysis, Molecular Electronics, Photonics, and Nanomedicine. Chem. Rev..

[30] Tomalia D.A., Baker H., Dewald J., Hall M., Kallos G., Martin S., Roeck J., Ryder J., Smith P. (1985). A new class of polymers–Starburst-dendritic macromolecules. Polymer J..

[31] Majoral J.P., Caminade A.M. (1999). Dendrimers containing heteroatoms (Si, P, B, Ge, or Bi). Chem. Rev..

[32] Caminade A.M. (2016). Inorganic dendrimers: Recent advances for catalysis, nanomaterials, and nanomedicine. Chem. Soc. Rev..

[33] Apartsin E.K., Knauer N., Kahlert U.D., Caminade A.-M. (2022). Amphiphilic Triazine-Phosphorus Metallodendrons Possessing Anti-Cancer Stem Cell Activity. Pharmaceutics.

[34] Zhao X.X., Loo S.C.J., Lee P.P.F., Tan T.T.Y., Chu C.K. (2010). Synthesis and cytotoxic activities of chloropyridylimineplatinum(II) and chloropyridyliminecopper(II) surface-functionalized poly(amidoamine) dendrimers. J. Inorg. Biochem..

[35] El Brahmi N., El Kazzouli S., Mignani S.M., Essassi E., Aubert G., Laurent R., Caminade A.M., Bousmina M.M., Cresteil T., Majoral J.P. (2013). Original Multivalent Copper(II)-Conjugated Phosphorus Dendrimers and Corresponding Mononuclear Copper(II) Complexes with Antitumoral Activities. Mol. Pharm..

[36] Mignani S.M., El Brahmi N., El Kazzouli S., Laurent R., Ladeira S., Caminade A.-M., Pedziwiatr-Werbicka E., Szewczyk E.M., Bryszewska M., Bousmina M.M. (2017). Original Multivalent Gold(III) and Dual Gold(III)-Copper(II) Conjugated Phosphorus Dendrimers as Potent Antitumoral and Antimicrobial Agents. Mol. Pharm..

[37] Sanz del Olmo N., Carloni R., Bajo A.M., Ortega P., Fattori A., Gomez R., Ottaviani M.F., Garcia-Gallego S., Cangiotti M., Javier de la Mata F. (2019). Insight into the antitumor activity of carbosilane Cu(ii)-metallodendrimers through their interaction with biological membrane models. Nanoscale.

[38] Mignani S., El Brahmi N., Eloy L., Poupon J., Nicolas V., Steinmetz A., El Kazzouli S., Bousmina M.M., Blanchard-Desce M., Caminade A.M. (2017). Anticancer copper(II) phosphorus dendrimers are potent proapoptotic Bax activators. Eur. J. Med. Chem..

[39] Ottaviani M.F., El Brahmi N., Cangiotti M., Coppola C., Buccella F., Cresteil T., Mignani S., Caminade A.M., Costes J.P., Majoral J.P. (2014). Comparative EPR studies of Cu(II)-conjugated phosphorous-dendrimers in the absence and presence of normal and cancer cells. RSC Adv..

[40] Alami O., Laurent R., Tasse M., Coppel Y., Colliere V., Bignon J., Majoral J.-P., El Kazzouli S., El Brahmi N., Caminade A.-M. (2023). Functionalization of graphene oxide surfaces with phosphorus dendrimer and dendron. FlatChem.

[41] Alami O., Laurent R., Tasse M., Coppel Y., Bignon J., El Kazzouli S., Majoral J.-P., El Brahmi N., Caminade A.-M. (2023). “Click” Chemistry for the Functionalization of Graphene Oxide with Phosphorus Dendrons: Synthesis, Characterization and Preliminary Biological Properties. Chem.-Eur. J..

[42] Sanz del Olmo N., Maroto-Diaz M., Gomez R., Ortega P., Cangiotti M., Ottaviani M.F., Javier de la Mata F. (2017). Carbosilane metallodendrimers based on copper (II) complexes: Synthesis, EPR characterization and anticancer activity. J. Inorg. Biochem..

[43] Carloni R., del Olmo N.S., Canonico B., Montanari M., Ciacci C., Ambrosi G., de la Mata F.J., Ottaviani M.F., Garcia-Gallego S. (2021). Elaborated study of Cu(II) carbosilane metallodendrimers bearing substituted iminopyridine moieties as antitumor agents. Eur. J. Med. Chem..

[44] Holota M., Michlewska S., Garcia-Gallego S., del Olmo N.S., Ortega P., Bryszewska M., de la Mata F.J., Ionov M. (2023). Combination of Copper Metallodendrimers with Conventional Antitumor Drugs to Combat Cancer in In Vitro Models. Int. J. Mol. Sci..

[45] Chen L., Mignani S., Caminade A.-M., Majoral J.-P. (2019). Metal-based phosphorus dendrimers as novel nanotherapeutic strategies to tackle cancers: A concise overview. Wiley Interdiscip. Rev.-Nanomed. Nanobiotechnol..

[46] Holota M., Magiera J., Michlewska S., Kubczak M., Sanz del Olmo N., Garcia-Gallego S., Ortega P., Javier de la Mata F., Ionov M., Bryszewska M. (2019). In Vitro Anticancer Properties of Copper Metallodendrimers. Biomolecules.

[47] Chen L., Fan Y., Qiu J., Laurent R., Li J., Bignon J., Mignani S., Caminade A.-M., Shi X., Majoral J.-P. (2020). Potent Anticancer Efficacy of First-In-Class Cu-II and Au-III Metaled Phosphorus Dendrons with Distinct Cell Death Pathways. Chem.-Eur. J..

[48] Sanz Del Olmo N., Holota M., Michlewska S., Gomez R., Ortega P., Ionov M., de la Mata F.J., Bryszewska M. (2020). Copper (II) Metallodendrimers Combined with Pro-Apoptotic siRNAs as a Promising Strategy Against Breast Cancer Cells. Pharmaceutics.

[49] Canonico B., Carloni R., Sanz del Olmo N., Papa S., Nasoni M.G., Fattori A., Cangiotti M., Javier de la Mata F., Ottaviani M.F., Garcia-Gallego S. (2020). Fine-Tuning the Interaction and Therapeutic Effect of Cu(II) Carbosilane Metallodendrimers in Cancer Cells: An In Vitro Electron Paramagnetic Resonance Study. Mol. Pharm..

[50] Launay N., Caminade A.M., Lahana R., Majoral J.P. (1994). A general synthetic strategy for neutral phosphorus-containing dendrimers. Angew. Chem.-Int. Edit. Engl..

[51] Slany M., Bardaji M., Casanove M.J., Caminade A.M., Majoral J.P., Chaudret B. (1995). Dendrimer surface-chemistry–Facile route to polyphosphines and their gold complexes. J. Am. Chem. Soc..

[52] Ouali A., Laurent R., Caminade A.M., Majoral J.P., Taillefer M. (2006). Enhanced catalytic properties of copper in O- and N-arylation and vinylation reactions, using phosphorus dendrimers as ligands. J. Am. Chem. Soc..

[53] Caminade A.-M., Hameau A., Majoral J.-P. (2016). The specific functionalization of cyclotriphosphazene for the synthesis of smart dendrimers. Dalton Trans..

[54] Huisgen R., Szeimies G., Moebius L. (1967). 1.3-Dipolare Cycloadditionen XXXII. Kinetik der Additionen organischer Azide an CC-Mehrfachbindungen. Chem. Ber..

[55] Kolb H.C., Finn M.G., Sharpless K.B. (2001). Click chemistry: Diverse chemical function from a few good reactions. Angew. Chem. Int. Ed..

[56] Zhou L.L., Roovers J. (1993). Synthesis of novel carbosilane dendritic macromolecules. Macromolecules.

[57] Llamazares C., del Olmo N.S., Ortega P., Gomez R., Soliveri J., de la Mata F.J., Garcia-Gallego S., Copa-Patino J.L. (2019). Antibacterial Effect of Carbosilane Metallodendrimers in Planktonic Cells of Gram-Positive and Gram-Negative Bacteria and *Staphylococcus aureus* Biofilm. Biomolecules.

[58] Llamazares C., Sanz del Olmo N., Soliveri J., de la Mata F.J., Copa-Patino J.L., Garcia-Gallego S. (2021). Insight on the Structure-to-Activity of Carbosilane Metallodendrimers in the Fight against *Staphylococcus aureus* Biofilms. Antibiotics.

[59] Lemaire V., Shemesh C.S., Rotte A. (2021). Pharmacology-based ranking of anti-cancer drugs to guide clinical development of cancer immunotherapy combinations. J. Exp. Clin. Cancer Res..

[60] Debela D.T., Muzazu S.G.Y., Heraro K.D., Ndalama M.T., Mesele B.W., Haile D.C., Kitui S.K., Manyazewal T. (2021). New approaches and procedures for cancer treatment: Current perspectives. SAGE Open Medicine.

[61] Caminade A.-M. (2022). Dendrimers, an Emerging Opportunity in Personalized Medicine?. J. Pers. Med..

